# Evolution of a zoonotic pathogen: investigating prophage diversity in enterohaemorrhagic *Escherichia coli* O157 by long-read sequencing

**DOI:** 10.1099/mgen.0.000096

**Published:** 2016-12-12

**Authors:** Sharif Shaaban, Lauren A. Cowley, Sean P. McAteer, Claire Jenkins, Timothy J. Dallman, James L. Bono, David L. Gally

**Affiliations:** ^1^​ Division of Infection and Immunity, The Roslin Institute and Royal (Dick) School of Veterinary Studies, University of Edinburgh, Easter Bush EH25 9RG, UK; ^2^​ Gastrointestinal Bacterial Reference Unit, 61 Colindale Avenue, Public Health England, London NW9 5EQ, UK; ^3^​ U.S. Meat Animal Research Center, Agricultural Research Service, U.S. Department of Agriculture, Clay Center, NE 68933-0166, USA

**Keywords:** Shiga toxin, bacteriophage, prophage, *Eschrichia coli* 0157

## Abstract

Enterohaemorrhagic *Escherichia coli* (EHEC) O157 is a zoonotic pathogen for which colonization of cattle and virulence in humans is associated with multiple horizontally acquired genes, the majority present in active or cryptic prophages. Our understanding of the evolution and phylogeny of EHEC O157 continues to develop primarily based on core genome analyses; however, such short-read sequences have limited value for the analysis of prophage content and its chromosomal location. In this study, we applied Single Molecule Real Time (SMRT) sequencing, using the Pacific Biosciences long-read sequencing platform, to isolates selected from the main sub-clusters of this clonal group. Prophage regions were extracted from these sequences and from published reference strains. Genome position and prophage diversity were analysed along with genetic content. Prophages could be assigned to clusters, with smaller prophages generally exhibiting less diversity and preferential loss of structural genes. Prophages encoding Shiga toxin (Stx) 2a and Stx1a were the most diverse, and more variable compared to prophages encoding Stx2c, further supporting the hypothesis that Stx2c-prophage integration was ancestral to acquisition of other Stx types. The concept that phage type (PT) 21/28 (Stx2a+, Stx2c+) strains evolved from PT32 (Stx2c+) was supported by analysis of strains with excised Stx-encoding prophages. Insertion sequence elements were over-represented in prophage sequences compared to the rest of the genome, showing integration in key genes such as *stx* and an excisionase, the latter potentially acting to capture the bacteriophage into the genome. Prophage profiling should allow more accurate prediction of the pathogenic potential of isolates.

## Data Summary

**Fig. 1.**
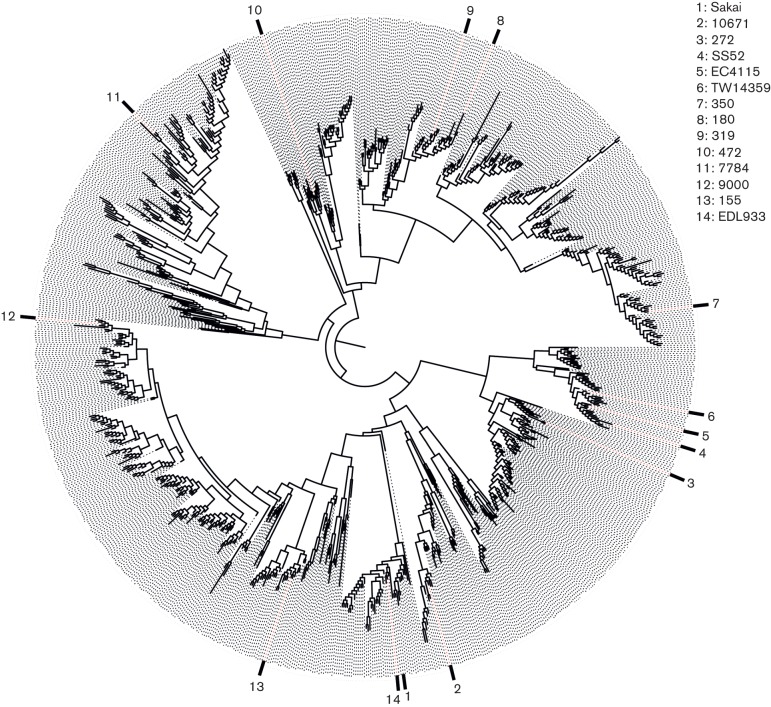
Maximum likelihood phylogeny of 956 isolates representing 22 805 SNPs across 3313 coding DNA sequences (CDSs) (2569 non-coding SNPs) with a total core genome size of 3 003 626 bp. The 14 isolates analysed in the present study are labelled. Their spread across the tree demonstrates the diversity of isolates analysed, which cover multiple lineages, geographical locations and PTs.

The code for the pipeline can be found at: https://github.com/SharifShaaban/PROPI. All the strain sequences used to generate Fig. 1 can be found under the BioProject ID PRJNA248042: https://www.ncbi.nlm.nih.gov/bioproject/?term=PRJNA248042. The information related to all the other strains used in this analysis can be found in Table 1. Two supplementary tables and two supplementary figures are available with the online Supplementary Material.

**Table 1.** Strains used in the analysis

Accession no.	Strain	BioSample ID	BioProject ID
CP018252	9000	SAMN05544760	PRJNA336330
CP018250	10671	SAMN05544761	PRJNA336330
CP018247	7784	SAMN05544762	PRJNA336330
CP018237	155	SAMN05544764	PRJNA336330
CP018243	350	SAMN05544765	PRJNA336330
CP018239	272	SAMN05544766	PRJNA336330
CP018245	472	SAMN05544767	PRJNA336330
CP018241	319	SAMN05544768	PRJNA336330
CP015832	180	SAMN05007044	PRJNA321984
NC_002695	Sakai	na	PRJNA57781
NC_011353	EC4115	SAMN02603441	PRJNA224116
NC_013008	TW14359	SAMN02604255	PRJNA224116
CP008957	EDL933	SAMN02905113	PRJNA253471
CP010304	SS52	SAMN03265100	PRJNA201344

na, Not applicable.

## Impact Statement

Enterohaemorrhagic *Escherichia coli* (EHEC) O157 : H7 strains pose a threat to human health and are usually acquired from ruminants, the environment or fresh produce. Recent whole genome sequencing based on short-read technologies has aided outbreak tracing and has provided insights into the evolution of this pathogen. However, these methods do not capture the genomic variation that underpins differences in zoonotic and pathogenic potential. This variation is, in part, driven by the acquisition of bacteriophages (phages), which contain many similar sequences requiring longer-read sequencing technologies to define their complete composition and position in the genome. This study has used Single Molecule Real Time (SMRT) sequencing, a long-read technique, to define the integrated phage sequences in an isolate set selected to represent the wide diversity of EHEC O157. We demonstrate that the most recent diversification correlates with acquisition of phages encoding specific types of Shiga toxin, responsible for the main damage and life-threatening consequences of EHEC in humans. Smaller phage regions have preferentially lost genes that allow phage production, and the density of insertion sequence elements in integrated phage regions supports their involvement in gene deletion and phage entrapment. Profiling of integrated phages will aid identification of virulent isolates from short-read sequencing currently being adopted more routinely in diagnostic laboratories.

## Introduction

The availability of additional sequences to compare with the first sequenced *Escherichia coli* genome, *E. coli* K12 MG1655, has highlighted how the evolution of this species is intimately associated with the integration of bacteriophages into the bacterial genome, and their subsequent entrapment, recombination and degradation as prophage regions (Ohnishi *et al.*, 2001; Shaikh & Tarr, 2003). The importance of specific prophages and their longer-term legacy are evident when considering the emergence of enterohaemorrhagic *E. coli* (EHEC) as a serious zoonotic pathogen (Hayashi *et al.*, 2001; Ogura *et al.*, 2009; Perna *et al.*, 2001). EHEC are defined by their capacity to cause bloody diarrhoea and, in a subset of cases, life-threatening haemolytic uraemic syndrome (Akashi *et al.*, 1994; Tarr *et al.*, 2005). EHEC O157 : H7 is one of the main serotypes associated with disease in Europe, North America and Asia, with infections usually originating from a ruminant reservoir, particularly cattle (Naylor *et al.*, 2005), although fresh-produce outbreaks are increasingly common (Lynch *et al.*, 2009; Marder *et al.*, 2014). Phylogenetic studies have established that *E. coli* O157 can be delineated into three main lineages, as well as nine clades (Eppinger *et al.*, 2011; Zhang *et al.*, 2007; Manning *et al.*, 2008). In the USA, clade 8 strains of lineage I/II are associated with more severe human disease (Manning *et al.*, 2008); while in the UK, the main human isolates reside in lineage I and clade 4/5 (Dallman *et al.*, 2015).

Human pathology is a direct and indirect result from the activity of Shiga toxin (Stx), a two-component toxin encoded on integrated bacteriophage and released with nascent bacteriophage following cell lysis (Tyler *et al.*, 2013). In addition, EHEC are defined by the expression of a type III secretion system that injects effector proteins into epithelial cells promoting colonization of the host (Kaper *et al.*, 2004; Tobe *et al.*, 2006). Furthermore, many of these injected effector proteins are encoded by prophage regions disseminated around the genome, forming part of a prophage regulatory network that is critical for the virulence of the organism (Tree *et al.*, 2009). Prophages are integral to the evolution of EHEC O157 genomes, but relatively few studies have investigated the potential prophage variability between the main lineages and sub-clusters of this clonal serotype. Seminal work on the prophage repertoire in the EHEC O157 Sakai strain was conducted by Asadulghani and colleagues, in which they demonstrated that bacteriophage diversity can be produced from this single EHEC O157 strain following SOS-based induction (Asadulghani *et al.*, 2009). However, comparative genomics of prophage regions in EHEC isolates is hampered by the inability of short-read sequencing to resolve the large number of repetitive and paralogous features indicative of prophage sequences.

Very recent research has provided insights into the evolution of EHEC O157 strains based on the sequencing of over 1000 isolates from human clinical cases and cattle hosts in the UK (Dallman *et al.*, 2015). This study demonstrated that the contemporary *E. coli* O157 clone emerged approximately 150 years ago from a strain harbouring a specific subtype of Stx: Stx2c. Only in the last 30–50 years was this subsequently followed by the independent acquisition of the Stx2a subtype by bacteriophage integration. Further, analysis of disease outcome indicated that more severe pathology was associated with isolates expressing Stx2a alone or in combination with Stx2c. As a consequence, it can be argued that the emergence of EHEC O157 as a serious human pathogen has coincided with the appearance of Stx2a-positive isolates in the ruminant reservoir (Dallman *et al.*, 2015).

Whilst phylogenetic analysis based on draft genomes continues to yield important insights into the epidemiology of EHEC O157, long-read sequencing platforms such as those developed by Pacific Biosciences (PacBio) and Oxford Nanopore are gaining traction, reducing the cost of complete genome sequencing and facilitating strain comparison of prophage regions (Anton *et al.*, 2015; McCarthy, 2010; Mikheyev & Tin, 2014). In the current study, we have investigated the prophage population present in 14 strains using PacBio RSII Single Molecule Real Time (SMRT) sequencing (nine isolates) and publically available genome sequences (five strains). The strains selected to examine the prophage diversity were chosen from the different lineages and main sub-clusters previously demonstrated for *E. coli* O157 based on core genome comparison (Dallman *et al.*, 2015). Sequences for whole prophage regions were identified, extracted, clustered and compared with respect to their annotated gene content. This work has demonstrated the stability of Stx2c-encoding prophages compared to Stx2a- and Stx1a-encoding prophages, and provided general insights into the evolution of prophages in the *E. coli* genome and their interplay with insertion sequence (IS) elements.

## Methods

### Sequences and sequencing.

For this analysis, 14 genome sequences were used. Five of these were publically available in the National Center for Biotechnology Information (NCBI) database, including genome sequences from the strains Sakai, EC4115, TW14359, EDL933 and the recently published super-shedding strain, SS52 (respective NCBI accession numbers: NC_002695, NC_011353, NC_013008, CP008957 and CP010304) (Table 1).

Sequencing of the nine isolates was conducted using a PacBio long-read sequencing RS II platform and carried out at the U. S. Department of Agriculture facility in Nebraska, USA. Qiagen Genomic-tip 100/G columns and a modified protocol, as previously described (Clawson *et al.*, 2009), were used to extract high molecular weight DNA. Using a g-TUBE (Corvaris), 10 µg DNA was sheared to a targeted size of 20 kb and concentrated using 0.45× volume of AMPure PB magnetic beads (Pacific Biosciences). Following the manufacturer’s protocol, 5 µg sheared DNA and the PacBio DNA SMRTbell Template Prep kit 1.0 were used to create the sequencing libraries. A BluePippin instrument (Sage Science) with the SMRTbell 15–20 kb setting was used to size select 10 kb or larger fragments. The library was bound with polymerase P5 and sequencing was conducted with the C3 chemistry and the 120 min data collection protocol.

### Assembly and annotation.

SMRT analysis was used to generate a fastq file from the PacBio reads, which were then error-corrected using PBcR with self-correction (Koren *et al.*, 2013). The Celera Assembler was used to assemble the longest 20× coverage of the corrected reads. The resulting contigs were improved using Quiver (Chin *et al.*, 2013) and annotation was conducted using a local instance of Do-It-Yourself Annotator (diya) (Stewart *et al.*, 2009). Geneious (Biomatters) was used to remove duplicated sequence from the 5′ and 3′ ends to generate the circularized chromosome. Initially, OriFinder was used to determine the origin of replication (Luo *et al.*, 2014) and the chromosome was reoriented using the origin as base number one. However, for visualization purposes, the genome orientation and first base were modified to match those of the main NCBI reference sequence (Sakai) using tools from the emboss tools suite (Rice *et al.*, 2000).

### Phylogenetic context.

To provide context for the selected strains, a phylogenetic analysis was conducted involving 943 isolates. These isolates were representatives from single linkage clusters defined at a 25 single nucleotide polymorphism (SNP) threshold from the Public Health England Shiga toxin-producing *E. coli* O157 genome collection available at the BioProject PRJNA248042 (Dallman *et al.*, 2015). As this approach requires Illumina reads, these were artificially created using wgsim from the whole genome sequences (Li *et al.*, 2009) for 13 of the 14 sequences analysed in this study (Sakai was already included). Illumina reads for all isolates (956 in total) were quality trimmed (Bolger *et al.*, 2014) and mapped to the reference EHEC O157 strain Sakai using bwa mem (Li & Durbin, 2010). SNPs were then identified using GATK2 (McKenna *et al.*, 2010) in unified genotyper mode. Core genome positions that had a high quality SNP (>90 % consensus, minimum depth 10×, GQ ≥30) in at least one strain were extracted and RaxML v8.17 (Stamatakis, 2014) used to derive the maximum likelihood phylogeny of the isolates under the gtrcat model of evolution (Fig. 1). This phylogeny demonstrated how the 14 selected strains for this analysis sample the diversity of EHEC O157.

### Prophage calling.

The PHAge Search Tool (phast) was used to extract prophage regions (Zhou *et al.*, 2011). Sequences were submitted using the phast urlapi. Prophage regions called by phast, regardless of size and quality score, were extracted from their respective genomes. Any two prophages that were separated by less than 4000 bp were joined and called as a single prophage, primarily as it was observed that gaps of this size and smaller were often due to IS elements. Prior testing was carried out on phast’s capacity to call prophage boundaries using prophages with known and studied insert sites, as well as the established Sakai prophages (SPs). The results obtained provided confidence in this approach; all SPs were found, while only two SP-like regions called as prophages. Due to the phast algorithm, prophage boundaries were often different to those defined for the related prophages in strain Sakai. phast uses an algorithm that calculates phage gene presence and distance, and the boundary is set based on short nucleotide repeats (when the phage contains an integrase), or when the distance between the phage genes is too wide (Zhou *et al.*, 2011). As a result, the extracted prophage sequences are sometimes extended beyond their physical integration sites. In addition, certain predicted prophages were found to overlap at their boundaries and these were not merged but included as separate prophages for the rest of the analyses. However, neither of these issues should have impacted on the main analyses presented.

Extracted prophages were annotated twice: once using the U. S. Department of Agriculture diya Glimmer-based pipeline, and the second time using Prokka (Seemann, 2014). The Prokka parameters given included a fasta amino acid database file obtained from the previously annotated whole genomes. The former annotation method led to more hypothetical proteins and provided a gene ID usable for gene ontology, while Prokka offered less hypothetical protein hits and more coding DNA sequence regions. Applying the diya annotation pipeline to the extracted prophage regions from the 14 strains resulted in a total of 9416 predicted gene products (2790 as hypothetical proteins), 523 of which were unique. This dataset had 863 unique RepIDs. These were provided as inputs for david (Huang *et al.*, 2009a, b), which recognized 428 of these IDs. However, the optimal functional classification result had 347 of these as singletons (only 81 genes were grouped). By comparison, Prokka annotation yielded 13 333 gene products, of which 2106 were hypothetical proteins, with 718 unique gene products.

The prophage GenBank (gbk) files obtained from the Prokka annotation were then modified so that annotated genes were given a colour flag based on their function. Functional groups were delimited as: metabolism and transport, structure, effector and virulence factors, recombination and replication, regulation, lysis, tRNA, and hypothetical or ambiguous genes. The group into which each gene was assigned was determined using a ‘key word’ classification (Table S1, available in the online Supplementary Material). The selection of the key words was determined by manual curation, supplemented by the results from the david analysis (data not shown). Only when a gene had a clear and studied specific function was it added to a group other than ‘hypothetical and ambiguous’.

### Prophage clustering.

Prophages were clustered based on gene homology that was obtained using GetHomologues version 1.0 (Contreras-Moreira & Vinuesa, 2013). When running the pangenome algorithms the parameter ‘-t 0’ was given in order to identify any gene even if it was only found in a single prophage. Paralogues were excluded using the ‘-e’ parameter. The cluster comparison tool of GetHomologues was run on the results to obtain binary (gene presence and absence) matrices for core and pangenome. The ‘–T’ flag was used to yield a parsimony pangenome tree (Fig. 3). This was done multiple times for different cut-offs of gene coverage and identity, jointly spanning from 70 to 95 in increments of five. The matrix applied for prophage clustering used 75 % identity and coverage cut-offs. This selection was based on the default coverage cut-off of the GetHomologues tool (75 %), which allows for potential gene truncation events to be taken into account. A high identity cut-off (e.g. 95 %) is not adequate considering the multiple potential prophage families being analysed, but a low identity cut-off (e.g. 50 %) would not provide enough discrimination. Therefore, a balance of 75 % nucleotide identity was selected, which allowed, in conjunction with gene co-occurrence, for appropriate clustering of related genes (Fig. S1), but did not yield too few clusters when compared to a stricter cut-off (<20 % difference, Fig. S2).

Hierarchical clustering was performed on the resulting binary matrix. The final number of clusters selected was determined based on the maximum Euclidean distance of any two members of a cluster. Multiple maximum Euclidean distance thresholds were chosen: 0 (as a fully identical gene content threshold), 1.5, 3.0, 4.5 (as the main comparative distance) and 6.0. The primary analysis distance of 4.5 was chosen as it translates to ~80 % blast sequence coverage and similarity. The other thresholds were used to establish the different levels of relationship between the clusters. With these thresholds, each prophage was assigned a code consisting of five values. These values indicated in which cluster the prophage was positioned at each threshold. These codes then served as a convenient and qualitative measure of prophage relationships (Table S2).

### Whole genome and prophage comparison.

**Fig. 2.**
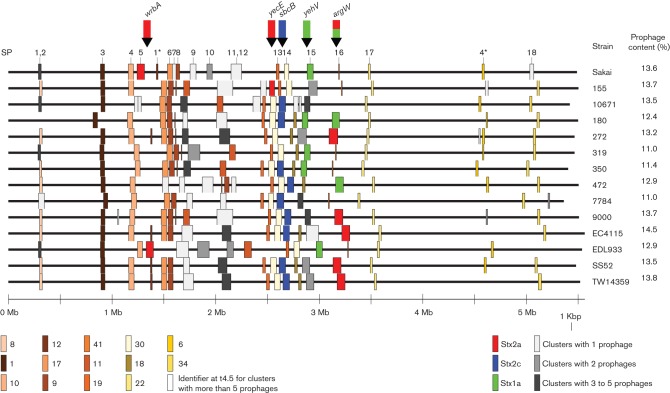
Easyfig whole chromosome alignment of 14 EHEC O157 sequences with prophage regions represented as coloured blocks. The numbers and positions of the previously defined SPs are provided (Hayashi *et al.*, 2001). Blocks of the same colour show levels of similarities that approximate 80 % blast coverage and identity (also referred to as t4.5 Euclidean distance). At the bottom left-hand side are the cluster identifiers for each coloured block. These identifiers indicate the clusters to which each prophage belongs at t4.5 (all cluster identifiers can be linked back to individual prophages using Table S2). Therefore, blocks of the same colour indicate prophages within the same cluster, except for grey blocks which indicate that the prophage belonged to a small cluster, and Stx-encoding prophages which are coloured by Stx subtype regardless of prophage similarity. Their associated insert sites are marked with arrows above the alignment, and colour coded based on observed Stx subtypes. The coloured blocks demonstrate the large population of prophages that is conserved across strains, as well as hotspots of variation for certain prophages. The overall percentage of prophage content for each isolate was calculated and is provided on the right-hand side.

Whole genome alignments were conducted with Easyfig (Sullivan *et al.*, 2011). The gbk files were modified so that prophages were represented as blocks with different colours based on the prophage code described above (Fig. 2). Easyfig alignments were also conducted on selected groups of prophages based on their clusters, inter-cluster relationships and Stx subtypes (Figs 4 and S1).

**Fig. 3.**
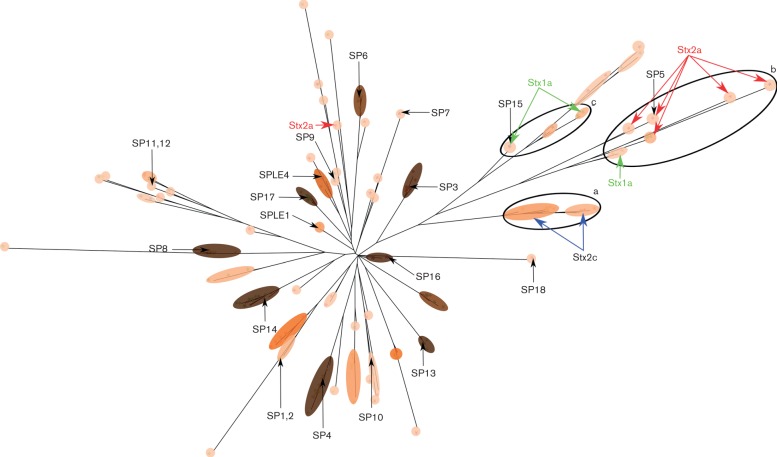
Midpoint rooted parsimony tree based on the gene content of 232 prophage sequences. SP numbers and Stx-encoding prophages are marked on the tree. The coloured overlay represents the clustering at t4.5 (approximately 80 % blast coverage and identity). The shade and colour of the overlay represents the number of prophages within these clusters, with darker ovals representing more isolates within a cluster. The size of the oval indicates how diverse members of a cluster are, with larger ovals being more diverse. This graphic indicates that the clusters that are present in the majority of the isolates analysed (dark brown, SPs 3, 4, 6, 7, 8, 13, 14 and 16) are often closely associated ‘tight clusters’ with little variation. Circles (labelled a–c) determine three groups of clusters that appear to relate to Stx-encoding prophages, with Stx2a prophages exhibiting significant diversity.

**Fig. 4.**
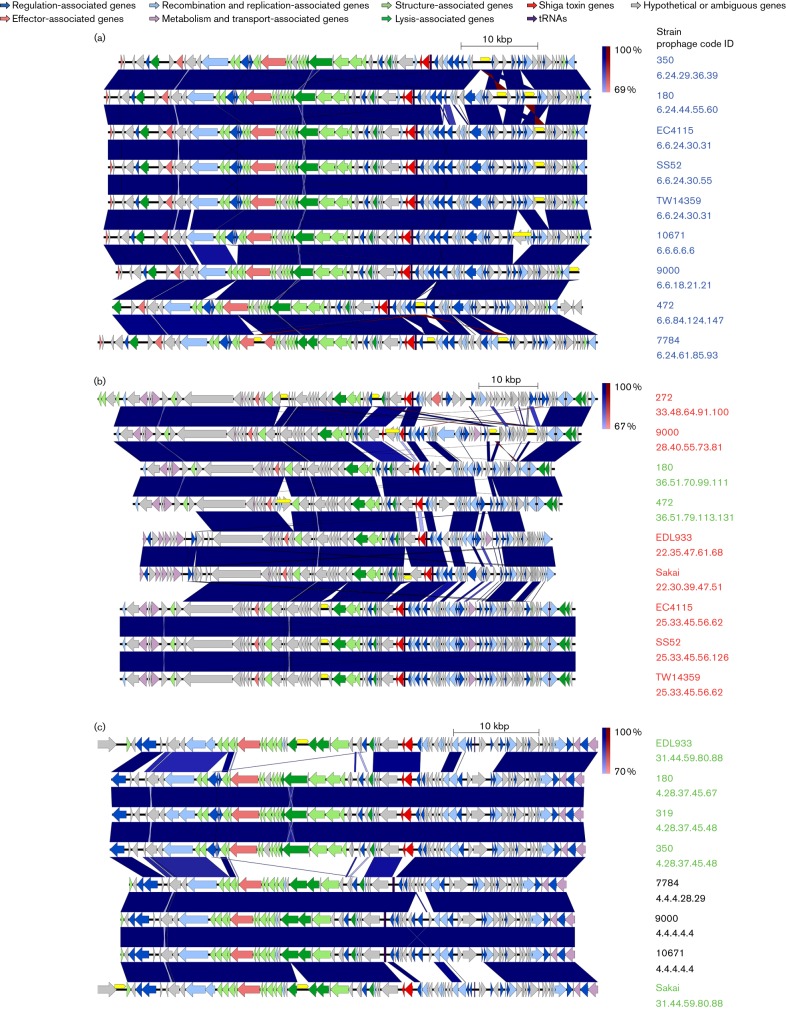
Easyfig alignment of the prophages contained in the circles (a–c) in Fig. 3. Genes are colour coded based on functional groups as indicated at the top of the figure. Prophage names are colour coded based on Stx content (blue, Stx2c; red, Stx2a; green, Stx1a; black, no Stx). The prophage code shown is based on Euclidean distance thresholds of 6.0, 4.5, 3.0, 1.5 and 0, and also indicates the relationship between the aligned prophages based on gene content. The alignments confirm the relationships shown in Fig. 3, with Stx2c-encoding prophages exhibiting a high degree of conservation, while Stx2a and Stx1a exhibit multiple subpopulations. IS elements are shown in yellow above the genes and are seen to interrupt multiple genes across the different prophages, including Stx2a in strain 9000 (centre alignment).

### Stx and IS calling.

The fasta sequences of all the genomes analysed were concatenated in a multi-fasta file and made into a blast database using blast+ version 2.2.29 (Altschul *et al.*, 1990; Camacho *et al.*, 2009). The Stx reference sequences obtained from Scheutz *et al.* (2012) were used as the query in a blastn comparison, with only the best scoring hits kept per Stx subtype.

ISs were identified using a blast database, extracted from IS Finder (Siguier *et al.*, 2006) (http://www-is.biotoul.fr), containing IS elements that originated from *E. coli* and *Shigella* (total of 119 sequences) (IS Finder accessed October 2015). IS regions were determined within prophages using the following blast parameters: ‘-evalue 1e-100 -best_hit_score_edge 0.0001 -best_hit_overhang 0.25 -outfmt 6’. In order to avoid repeated IS hits from closely related IS sequences, this was followed by the filtering of hits with a minimum match length of over 700 bps, and hits with the same starting or ending position were collapsed to the one with the highest bit score. These IS regions were given a colour flag in the prophage gbk files. However, it should be noted that the single IS elements highlighted in this study usually contained two to three individual genes.

### Gene frequencies and graphs.

**Fig. 5.**
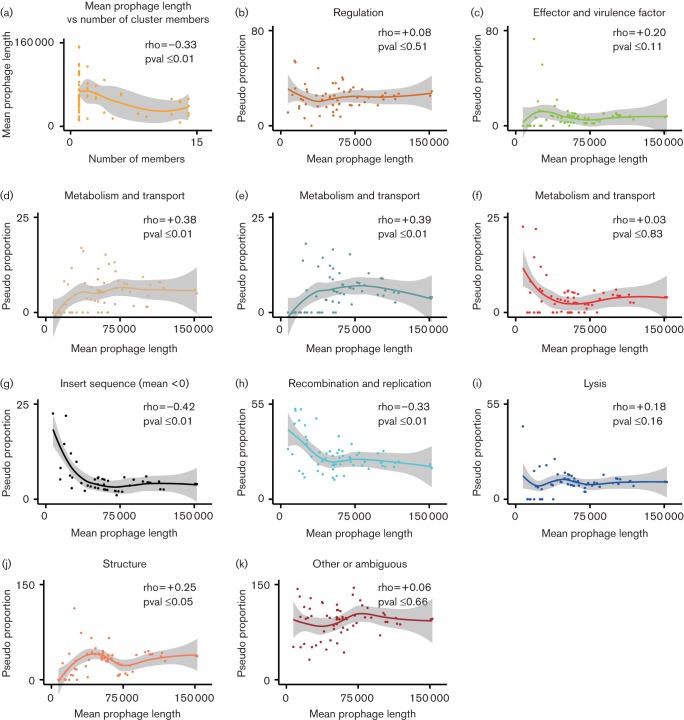
(a) The mean prophage length of a cluster over its number of members, indicating a statistically significant association with more members for shorter prophage sequences (*P*≤0.01). (b–k) All plots represent the proportion of genes from a functional group in a cluster over its mean prophage length. (b, c, f, i and k) These show no statistical significance between their gene function groups and mean prophage length. (d, e, g, h and j) These show statistical significance relating these particular function groups to the mean prophage length of clusters (*P*≤0.05).

Based on the annotated functional groupings described in the 'Prophage calling' section of Methods, gene content against the mean length of prophages was plotted in R with ggplot2 (Wickham, 2009). The mean number of genes for each annotated function within each cluster at a Euclidean distance of 4.5 was calculated. A multiplication step was then applied to this so that values could be computed correctly and easily in Linux. For this, the mean gene number was multiplied by the maximum prophage length in bp. The pseudo-proportions were then determined by dividing the result by the mean prophage length of the specific cluster (bp). These were then plotted in R as a scatter plot and a Loess line drawn to visualize the line of best fit. To determine the significance of these trends a Rho Spearman rank test was conducted in R (Fig. 5).

### Selecting spontaneous-cured lysogens using a temperature sensitive plasmid with TcR under CI control.

The tetracycline resistance gene with native Ribosome Binding Site (RBS), but without a promoter, was amplified from pBR322 using primers Nt-pTOF24-TcR (5′-aaactgcagagatcttaacgcagtcaggcaccgtgtatg-3’) and Ct-pTOF24-TcR (5′-aaactcgagcgaggtgccgccggcttccattca-3′) and cloned into pTOF24 ([Bibr R28][Bibr R28]), following restriction with BglII and XhoI. Screening was carried out on chloramphenicol resistant (CmR) colonies with the same primers; the bacteria were still sensitive to tetracycline at this point because no promoter was cloned. The oL/pL promoters from the relevant Stx-encoding prophage were then amplified with primers (as below) from a lysogen and cloned into pTOF–TcR with 5′-PstI and 3′-BglII after an In-Fusion kit (Clontech) cloning step for the amplicons. Selection was for CmR. The primers for the Stx2c-prophage were 5′-Sp5pL-PstI-IF (5′-gtctcggtacccgacctgcagcctctcgcccaaaaaaacacataac-3′) and 3′-Sp5pL-BglII-IF (5′-tgcctgactgcgttaagatcttgccagtctgttccatttggc ttcc-3′). The primers used for the Stx2a-prophage were 5′-560stx2pL-PstI-IF (5′-gtctcggtacccgacctgcagctttgcctcacgttcgc ccacc-3′) and 3′-560stx2pL-BglII-IF (5′-tgcctgactgcgttaagatcttcctgctgacgatgataataatg-3′) with the constructs used on isolate 9000. A check was then carried out to determine whether the level of TcR was lower in a lysogen compared to a non-lysogen background. When spontaneous excision of the phage occurs, the isolate reverts to a higher level of TcR as pL is activated due to a lack of CI repression. Lysogen curing was verified by PCRs across insert junctions. The final step was to remove pTOF24-oL/pL-TcR from the spontaneous lysogen-cured isolate based on growth under restrictive temperature conditions, without antibiotic.

## Results

### Prophage content and location in *E. coli* O157 genomes

The strains selected for prophage analysis in this study are highlighted in Fig. 1 and sampled the wide diversity of the phylogeny, including lineages and susceptibility to a panel of typing phages that define phage type (PT). We detected 232 prophages and extracted their sequences. The positions and sizes of the prophages in their respective genomes are illustrated in Fig. 2, which indicates the more stable prophage populations with consistent locations across the genomes. Prophage size ranged from 6126 to 152 606 bp and the estimated prophage content per genome ranged from 11.0 to 14.5 % (Fig. 2). Many of the prophages matched those originally designated as SPs (Hayashi *et al.*, 2001). Prophage distribution across the chromosome was biased to regions between bp 800 000 and 3 600 000, with ~83 % of all prophages found within these boundaries. All Stx-encoding prophages were found in previously documented insert sites (Shaikh & Tarr, 2003): prophages encoding Stx2c were found only in *sbcB*; while those encoding Stx2a were found in multiple insert sites – *wrbA*, *argW*, *yecE*; and Stx1a-encoding prophages were detected in *yehV* and *argW*. The majority of strains contained only one or two Stx-encoding prophages.

### Prophage clustering

The extracted prophages were clustered according to related gene content. There was no single gene common to all the analysed prophages at a cut-off of 75 % coverage and nucleotide identity; this reflects the wide diversity of temperate phage backgrounds integrated into the *E. coli* O157 genome. Using the outputted binary matrix and with selected Euclidean distance thresholds of 6.0, 4.5, 3.0, 1.5 and 0, hierarchical clustering yielded 42, 63, 86, 128 and 151 clusters, respectively (full clustering in Table S1). This indicated that within our 232 prophages there were 151 different individual prophages based on gene content alone. Based on subsequent alignments (Figs 4 and S1), the majority of these clusters appeared adequate, although difficulties could arise when analysing the smaller prophages (6000–9000 bp) in this dataset, because their relatively low gene content results in less discriminatory power. The main benefit of this approach was that the relatedness of different prophages and their clusters could be visualized at different relative scales.

The midpoint rooted parsimony-based pangenomic tree obtained from GetHomologues is shown in Fig. 3, with clusters shaded within a Euclidean distance threshold of 4.5 (t4.5). Multiple cluster types were observed including singletons, clusters with only a single prophage (SP18 and SP7). Typically, these were either at the start or at the end of a branch, indicating that they either lacked the genes defining the other cluster(s) on that branch if at the start of the branch, or possessed additional gene content to the other branch members if at the end of the branch. There were also specific singletons that appeared to be prophages integrated within other prophages. There were prophage clusters with representatives in nearly all the strains analysed (indicated by the darker shading in Fig. 3) and these were often tightly grouped, for example SP13, SP16 and SP17. Other clusters had intermediate representation in the analysed strains and these could be more divergent (SP10 and SP11, 12).

### Stx-encoding prophage comparison

All but one (155 Stx2a) of the Stx-encoding prophages were present on the same branch off the main root of the prophage tree (Fig. 3). These diverged into prophage sub-clusters that associated well with specific Stx subtypes. It was noted that not all prophages within these branches necessarily carried Stx genes. Therefore, Stx-negative prophages could be found with very similar gene content to Stx-encoding prophages. Stx2a-encoding prophages were the most divergent, containing five sub-clusters (Fig. 3, circle b) based on only seven prophages. By comparison, Stx1a-encoding prophages were also relatively divergent with three sub-clusters from seven prophages (Fig. 3, circles b and c). Finally, Stx2c-encoding prophages exhibited the least diversity, with only two sub-clusters based on nine prophages (Fig. 3, circle a).

The main prophage clusters associated with *stx* were further compared by Easyfig sequence alignment (Fig. 4). The least sequence and gene content diversity was shown by the Stx2c-encoding prophages (Fig. 4a), where the main variation was associated with IS element insertion (designated by yellow blocks in Fig. 4). The prophages were assigned a code based on the clustering at the different Euclidean distances (Fig. 4). The Stx2c-encoding prophages were all within the same t6.0 cluster and two t4.5 clusters. By contrast the Stx2a-encoding prophages were more variable and the prophage groupings also contained two Stx1a-encoding prophages (Fig. 4b). There were four t6.0 and five t4.5 in relation to *stx2a* alone. The American reference genomes EC4115, SS52 and TW14359 appeared to have near identical Stx2a-encoding prophages (identical up to t1.5), while the Sakai and EDL933 Stx2a-encoding prophages were related to each other up to t6.0. The Stx2a-encoding prophages demonstrated high levels of variation in the final third of the prophage, often starting adjacent to the *stx* locus. The demonstration of two Stx1a-encoding prophages in these sub-clusters is indicative of acquisition of a different Stx subtype by a similar prophage background. This aligns with their insertion in *argW*, which occurs for a subset of the Stx2a-encoding prophages, and shows that the prophage background is more relevant to the insert site than *stx* content.

The remaining Stx1a-prophages clustered separately, consisting of two t6.0 and two t4.5 clusters (Fig. 4c). The first subtype was present in EDL933 and Sakai, while the second was in UK isolates 180, 319 and 350. A related third subtype was Stx negative and more closely related to the sub-cluster present in EDL933 and Sakai.

### Integration of a Stx2a-encoding prophage can confer a switch from PT32 to P21/28

A common PT, PT21/28, associated with human infection in the UK, usually contains both Stx2a- and Stx2c-encoding prophages (Dallman *et al.*, 2015). By contrast, a phylogenetically close sub-cluster of PT32 contains predominantly Stx2c-encoding prophages only (Matthews *et al.*, 2013; Dallman *et al.*, 2015). We therefore tested whether excision of the Stx-encoding prophages from PT21/28 isolate 9000 (Fig. 1, Table 2) could alter the PT, which is based on resistance or susceptibility to a characterized set of T4 and T7 lytic phages (Cowley *et al.*, 2015). To generate these strains, we took advantage of the fact that lambda-like prophages continually express CI to remain in the lysogenic state. We reasoned that if we placed the prophage specific CI-repressed promotor in front of an antibiotic-resistance gene (in this case tetracycline resistance) on a plasmid transformed into isolate 9000 (PT21/28), then we would only get tetracycline resistance if the relevant prophage excised, relieving CI-based repression of the tetracycline resistance. This system worked well for the P_R_ promoters and we were able to routinely obtain spontaneous excision of the Stx2a-encoding prophage from isolate 9000. By contrast, a 9000 variant with a completely excised Stx2c-prophage was not obtained; instead partial deletions occurred that were missing the main phage lysis/lysogeny regulatory proteins and the *stx*2c genes (data not shown). A double deletion was attempted twice, once by first selecting for Stx2a-prophage excision and then Stx2c-prophage excision (partial), and the second time in the reverse order. Phage typing of the resultant strains demonstrated that any strain that had excised the Stx2a-prophage had a PT32 designation, rather than the PT21/28 of the parent strain (Table 2).

**Table 2.** The PT of the original strain 9000 and, when modified, a description of the modifications

Strain	Description	PT
9000	Original PT21/28 IPRAVE isolate, Stx2a and Stx2c	21/28
9000–2	9000 with Stx2c phage partly deleted	21/28
9000–3	9000 with Stx2a phage entirely deleted	32
9000–4	9000–2 with Stx2a phage entirely deleted	32
9000–5	9000–3 with Stx2c phage partly deleted	32

### IS elements

IS activity is critical to the evolution of individual isolates. For example, isolate 9000 had an ISec8 (IS*66* family) within the *stx*2a subunit A gene that is likely to disrupt Stx2a production (Fig. 4b). The same isolate also had an IS*629* (IS*3* family) in a flanking excisionase of the Stx2c-encoding prophage that could impact on prophage induction (Fig. 4a). Other IS activity can be seen in Figs 4 (and S1) clearly delineated by gaps in the blast alignment. IS elements appear in regions of recombination and change, with a bias towards prophage regions (Ooka *et al.*, 2009). We found 277 IS elements in the prophage regions of our strains, with 401 IS elements across the whole chromosome regions; the latter based on the same method of extraction as for prophage regions but using whole genome sequences (this can, however, include highly similar IS blast hits in the same location). This translates to 69.1 % of IS elements in 12.9 % of the whole genomes (prophage regions).

### Statistical analysis of prophage content and size

We hypothesized that as these *E. coli* genomes contained prophages that were acquired at different times, older prophages will have been under the evolutionary pressure of the host isolate for longer and should be more host adapted. Certain SPs are cryptic, i.e. can no longer produce viable bacteriophages (Asadulghani *et al.*, 2009), but presumably this process from productive prophage to cryptic prophage is a continuum, in which genes useful to the host bacterium may be retained and those specific to bacteriophage production are lost. We investigated this in two ways; in the first we asked whether there was a correlation between the mean length (bp) of a prophage cluster (defined at t4.5) and frequency in the analysed strains. As indicated in Fig. 5(a), shorter prophages are more likely to be associated with a greater number of strains (*P*≤0.01). The second approach examined the correlation between functional gene content and the mean length (bp) of prophage clusters at t4.5 (Fig. 5b–k).

From the analysis, the prophage sequences yielded 13 333 potential proteins, of which 2106 were hypothetical. These were sorted into 718 unique gene products that were used for functional groupings (Table 3), and the key words used for functional assignments can be found in Table S1. Genes functionally annotated as regulatory (Fig. 5b), effectors and virulence factors (Fig. 5c), lytic (Fig. 5i) and ‘other’ (Fig. 5k) showed no significant correlation with prophage length. IS element content was then examined in all prophages and no correlation was evident (Fig. 5f). However, we noted that IS elements were completely absent from a subset of prophage clusters, so we examined whether the proportion of IS element content correlated with prophage length for clusters infected with at least one IS element (Fig. 5g). In this case there was a correlation, with shorter prophages containing a relatively higher proportion of IS elements. A similar correlation was seen for recombination and replication-associated genes in all prophages (Fig. 5h). The proportions of three functional groupings showed evidence of a reduction as the mean prophage length shortened. These were metabolism and transport (Fig. 5d), tRNAs (Fig. 5e) and structural, predominately phage tail, head and baseplate, genes (Fig. 5j).

## Discussion

This study aimed to analyse prophage regions extracted from *E. coli* O157 : H7 strains representative of the main clusters found in the extensive phylogeny presented by Dallman *et al.* (2015). This phylogeny was based on core sequence analysis, but does not provide an indication of how the accessory genome, in particular prophage composition, may vary. Central to EHEC O157 virulence in humans is the subtype of Stx and control of Stx production, intrinsically linked to the prophage in which it is encoded. The original model of Stx acquisition hypothesized that Stx1-encoding prophages were the first to be acquired by EHEC O157, followed by Stx2-encoding prophages. However, due to the presence of differing Stx subtypes, the situation is more complex: a recent study looking at the phylogeny of over 1000 EHEC O157 isolate was suggestive that the original acquisition of Stx2c-encoding prophages by EHEC O157 occurred about 150–175 years ago, followed by later acquisition of Stx2a and Stx1a (Dallman *et al.*, 2015). However, this analysis was limited by the drawbacks of short-read sequencing. In our current study, we have been able to extract and examine a large number of prophage sequences from 14 strains, including those encoding Stx. Stx2c-encoding prophages were found to be less variable in their gene content and sequence similarity across strains from different geographical locations and lineages, compared to more diverse Stx1a- and Stx2a-encoding prophages.

The core genome phylogeny study (Dallman *et al.*, 2015) indicated that PT21/28, a PT associated with the majority of serious human EHEC infections over the last decade, emerged from a PT32 progenitor. In the present study, we demonstrated that excision of a Stx2a-encoding prophage from a PT21/28 isolate (9000) results in the isolate being defined as PT32. This is due to resistance to typing phages 6 and 13, which is a facet of the PT21/28 phenotype, but not the PT32 phenotype (Cowley *et al.*, 2015). It should be noted that clades of PT32 exist containing both Stx2a- and Stx2c-encoding prophages (Dallman *et al.*, 2015), indicating that conversion with a Stx2a-encoding prophage does not necessarily always lead to an altered lytic phage susceptibility. This presumably reflects the specific cluster of Stx2a-prophage involved in the lysogenization with a subset causing this typing transition. Our clustering of Stx2a-encoding prophages shows these to be one of the most diverse prophages present in the *E. coli* O157 clonal complex, with variation in the regulatory regions underpinning the different Stx2a-encoding prophage clusters. Numerous reports in the literature have observed these multiple Stx2a-encoding prophage subtypes (Ogura *et al.*, 2009; Yin *et al.*, 2015), with one paper classifying these subtypes using PCRs across the prophage regulatory region (Ogura *et al.*, 2015). The Stx2a-encoding prophages presented in our analyses can generally be classified into their groupings (alpha to zeta) by *in silico* approaches using primer recognition (data not shown). However, the PCR method places Stx2a-encoding prophages of strains EC4115, SS52, TW14359 and EDL933 in the same group, while Euclidean clustering groups the ones of EDL933 closer to Sakai, because it also takes into account differences outside the regulatory region. In addition, the Stx2a-encoding prophage of isolate 155 did not classify into any of their predefined groups, including ‘untypable’; thus, clearly demonstrating the marked difference in that particular Stx2a-encoding prophage compared to others so far studied.

Our methods also found two Stx1a-encoding prophages in isolates 180 and 472, which clustered closely to those usually encoding Stx2a. Both of these were defined as gamma type (Ogura *et al.*, 2015) (in one case both primers were found, while in another only one was found), suggesting that different Stx subtypes have been acquired by closely related temperate bacteriophage types, although whether this can impact on the pathogenic potential of the isolate is unknown. Many more Stx2a- and other Stx-encoding prophage sequences are required, along with metadata around the associated infections, in order to fully analyse the implications of the different Stx-encoding prophage subtypes. A longer-term objective of our work is to develop methods to define prophage content from short-read sequence data in order to help predict pathogenic potential. However, this will demand a deeper understanding of the current prophage populations generated by long-read sequencing methods, and more access to disease-severity data as well as to other epidemiological traits associated with the sequenced isolates.

A seminal research paper for EHEC was published in 2009 (Asadulghani *et al.*, 2009), examining the Sakai strain prophage content and capacity of such regions to be circularized and form infective phage particles. It was observed that many of the prophage regions contained mutations and deletions that should inhibit excision/replication and/or phage production. Of the 18 SPs, 9 were shown to be excisable either spontaneously or following mitomycin induction, whereas 6 SPs were packaged in a DNAse-resistant manner and 4 prophage regions could be transduced into *E. coli* K12. Although this indicated that several prophages considered cryptic could be excised, it was also apparent that nine showed no evidence of excision. From our study three of these ‘fixed’ prophages were present in most of our analysed strains at a high level of similarity (SP3, SP8 and SP17), three others (SP1, SP2 and SP16) were limited in diversity and only found in subsets of our strains, while the remaining three (SP11, SP12 and SP18) were only found in Sakai. Conversely, we have shown that the most variation in prophage regions was present in excisable prophages, which may be due to increased recombination activity during replication. Specifically, in the Asadulghani *et al.* (2009) study, generation of a hybrid Stx1-encoding prophage was identified, based on the SP5 background, indicating exchange of the Stx1-encoding region into the SP5 prophage. As previously stated, we noted that in our study, two of the UK isolates (180 and 472) contained SP5-like prophages, but encoded Stx1a (Fig. 4b), supporting the potential occurrence of this recombination in wild-type isolates. It was also apparent that the majority of variation present in Stx2a-encoding prophages (SP5-like) occurred in a region that encodes Stx2a regulatory and accessory genes, but distinct from structural and lytic genes (Fig. 4b).

We propose a model of prophage entrapment and ‘fixing’ based on the activity of IS elements in the *E. coli* O157 genome. This would then be followed by attrition/loss of specific genes that are no longer of value to the bacterium or phage, because it subsequently cannot produce viable bacteriophage (at least without helper phage activity). In support of IS ‘entrapment’, we observed IS insertion into the excisionase (*xis*) of the Stx2c-encoding prophage of isolate 9000, which would be expected to prevent prophage excision from the genome. It was also evident from our work that the Stx2c-prophage does not excise cleanly from isolate 9000, although the molecular basis for this requires elucidation. In the Asadulghani *et al.* (2009) study, they also noted that several prophages (including SP4 and SP14) contained truncated excisionases. It is proposed that combinatorial IS activity will result in rearrangements and deletions: we observed that prophage regions in our strains were biased for IS insertions, as has been noted previously (Ooka *et al.*, 2009). Alignment of the majority of the prophage regions (Figs 4 and S1) indicated that much of the variation in conserved prophages was due to IS integration.

In our study, we carried out functional gene annotation of the prophage regions to assess whether particular functional groups were retained or lost as prophages co-evolved with the bacterium. Specifically, we hypothesized that shorter prophages may represent elements that have been trapped for longer in the genome and may have undergone more gene deletion events. In support of this, there was a negative correlation between prophage length (bp) and their frequency in the strains, indicating that shorter prophages were more commonly found in the majority of strains. In addition, certain functional groups were preferentially lost in relation to prophage size, including structural genes that are predominately phage associated, such as head, tail and baseplate genes. This agrees with the concept that these genes may no longer be of value once the prophage is ‘fixed’. Lysis genes showed no significant trend, which may reflect a requirement for their maintenance or simply a need for more sequences, as the number of such genes was limited. Effectors and virulence factors showed no significant trend with size. Functional groups that were preferentially maintained included recombination and replication, which in conjunction with the trend observed for maintenance and spread of IS elements is indicative of the value of these genes in the bacterium. IS elements and recombinases generate diversity that may be a critical factor for their retention. The strains we analysed were from different geographical locations and collected at different times, yet contained many prophages that were nearly identical between all the strains. Therefore, while we are viewing only evolutionary snapshots, it would appear that this evolution follows a model where genes get either entrapped or lost from an isolate, and this then becomes a key representative isolate within a population.

A further observation from our study was the presence of an IS element within the Stx2a A subunit of isolate 9000. A number of studies have demonstrated similar integration and excision into and out of *stx* genes (Asano *et al.*, 2013; Park *et al.*, 2013; Toro *et al.*, 2015). Under certain conditions the loss of Stx prophages and Stx activity has been demonstrated, indicating that Stx activity may be a negative factor in certain cases and an advantage in others (Park *et al.*, 2013). IS elements, therefore, have the potential to provide heterogeneity within populations, further driving their maintenance in, or close to, strongly selective loci. Further work is required to examine IS element stability, and the effects on gene expression.

In summary, the identified prophage population is diverse but can be classified, potentially allowing clusters to be called from short-read sequencing data. By continuing to expand our prophage database from long-read approaches, we aim to be able to provide prophage profiles (similar to Fig. 2) that can have predictive value when coupled with epidemiological metadata. In this way, we aim to extract both ‘core’ SNP data and accessory prophage data from reads for diagnostic and public-health benefit.

**Table 3.** The number of gene products falling in each of the functional categories established in Methods The ‘All gene products’ column enumerates all combined gene products including repeats, while the ‘Unique gene products’ column does not include duplicates.

Functional group	All gene products	Unique gene products
Regulation	1500	136
Effector and virulence factors	564	45
Metabolism and transport	302	44
tRNA	429	429
Recombination and replication	1629	109
Lysis	686	28
Structure	2174	108
Hypothetical or ambiguous	6001	228

## Supplementary Data

999 Supplementary File 1
