# Supplementary material for: Evolution of a zoonotic pathogen: investigating prophage diversity in enterohaemorrhagic Escherichia coli O157 by long-read sequencing
Source: Microb Genom. 2016 Dec 12;2(12):e000096. doi: 10.1099/mgen.0.000096 (PMC5359411; doi:10.1099/mgen.0.000096)

SUPPLEMENTARY MATERIAL

Supplementary Table 1: A list of all the key words used to classify gene products into their respective gene groups.

Regulation	Recombination and Replication	Effector and Virulence Factors	Metabolism and Transport	Structure	Lysis	tRNA	Stx
anti-repressor	excisionase	effector	transport	scaffold	holin	tRNA	shiga
anti-termination	integrase	T3S	metabolism	capsid	lysozyme		
antirepressor	transposase	virulence	hydrolase	terminase	DNA packaging		
antitermination	resolvase	viral	synthesis	structural	DNA-packaging		
antiterminator	recombination	Type III	glycosidase	tail	portal		
anti-terminator	recombinase	Intimin	synthase	assembly	DNA injection		
cold-shock	recombinatory	espA	synthetase	head	DNA transfer		
cold shock	replication	multidrug	ribosomal	structure	lysis		
heat-shock	replicative	tape measure	thiolase	fimbrial	release		
heat shock	Insertion element	toxin	lyase	flagellar	racemase		
regulation	invertase	Eae protein	pump	baseplate	endopeptidase		
regulator	ninB	EscD	aminotransferase	filament	carboxypeptidase		
regulatory	ninG	EspF	amino-transferase	lipid	host killing		
helicase	ninH	enterohemolysin	protease	lipoprotein	endolysin		
antibiotic resistance	restriction	Lom protein	phosphate reductase		endo-lysin		
repressor	Kil protein	NleB	uptake		lisogenization		
zinc	host specificity	NleA8	periplasmic		lytic		
sensor	IS	secreted	sortase				
dipeptidase	DnaB	secretion	permease				
deacetylase	DicF	SepL protein	Bifunctional Protein PutA				
5-dehydrogenase	DNA primase		Bifunctional protein TrpGD				
glucosamine kinase	DNA polymerase		kinase				
glucosamine-kinase	transposition						
dna-binding	protein RecT						
dna binding	RecT protein						
methylase	TnpA						
sulfurtransferase	Cl protein						
acetyltransferase	cspa						
control	cytochrome						
ATP-binding							
ATP binding							
Cro							
Ren protein							
CII							
inhibitor							
activator							
derepression							

protein Sxy
sensing
sensor
Tir chaperone
Tir-cytoskeleton
Tir cytoskeleton
Tir protein
EspD

Supplementary Table 2: The complete clustering of all 232 prophages isolated within this analysis, with their strain of origin, their coordinates, and their length, as well as their cluster identifiers at distances to t6, t4.5, t3, t1.5, and t0.

Name	t6.0	t4.5	t3.0	t1.5	t0
10671_1220143_1258014_37871	12	14	14	14	15
10671_1254359_1285374_31015	10	59	78	112	129
10671_1465200_1518384_53184	4	17	17	45	49
10671_1532297_1578828_46531	9	9	9	29	30
10671_1665440_1731797_66357	24	29	71	120	142
10671_2018314_2075651_57337	11	11	11	11	114
10671_2366813_2436697_69884	17	21	23	24	25
10671_2460554_2485425_24871	15	19	20	21	22
10671_2536389_2589863_53474	25	30	50	69	74
10671_2616830_2680503_63673	3	13	80	114	133
10671_2760937_2802702_41765	14	48	56	77	84
10671_2812651_2831280_18629	2	49	57	78	85
10671_2863355_2916613_53258	20	38	42	59	64
10671_293754_320255_26501	8	16	16	18	19
10671_3449355_3468595_19240	18	22	24	27	108
10671_4549287_4569828_20541	6	6	6	6	7
10671_5040752_5062519_21767	27	34	37	47	52
10671_896000_934586_38586	1	1	1	1	1
155_1163777_1213353_49576	10	10	10	128	151
155_1479855_1534365_54510	4	17	17	90	101
155_1548278_1594812_46534	9	9	9	9	102
155_1618824_1624951_6127	2	41	48	66	71
155_1695832_1753827_57995	11	11	11	49	54
155_2019837_2170728_150891	13	25	28	35	38
155_2440924_2493826_52902	42	63	85	125	147
155_2524707_2574378_49671	38	57	76	110	127
155_2611109_2635976_24867	15	19	20	21	22
155_2687007_2741812_54805	25	30	83	121	143
155_2904189_2985879_81690	28	35	39	53	58
155_302577_327363_24786	8	8	38	93	105
155_3219318_3227870_8552	2	41	67	92	104
155_3508466_3528926_20460	18	22	24	67	72
155_4612202_4638929_26727	6	55	72	105	121

155_5112304_5134072_21768	27	34	37	103	117
155_894662_933247_38585	1	1	1	1	1
180_1151764_1202516_50752	10	10	10	88	97
180_1470329_1523352_53023	4	17	17	33	36
180_1537265_1583797_46532	9	9	9	9	10
180_1608572_1614699_6127	2	41	48	66	71
180_1688569_1733919_45350	31	56	73	106	122
180_2012340_2115143_102803	23	28	31	39	42
180_2452362_2477236_24874	15	19	20	21	22
180_2528266_2583406_55140	25	30	46	63	68
180_2608633_2672239_63606	3	3	61	82	90
180_2759892_2783970_24078	14	18	19	34	37
180_2841758_2897184_55426	20	24	27	32	35
180_3130862_3201124_70262	7	7	75	109	126
180_3483227_3502480_19253	18	22	24	27	28
180_4620508_4632339_11831	6	6	21	22	23
180_5111467_5133234_21767	27	34	37	47	52
180_819388_861917_42529	1	1	47	64	69
272_1165145_1215885_50740	10	10	10	10	137
272_1376569_1390740_14171	2	12	12	119	141
272_1482386_1534249_51863	4	17	17	17	18
272_1548162_1592359_44197	9	9	26	30	32
272_1616375_1622502_6127	2	41	48	66	71
272_1682627_1740665_58038	24	29	32	40	44
272_2042264_2141124_98860	5	5	5	5	6
272_2476623_2501497_24874	15	19	20	21	22
272_2552529_2605871_53342	25	30	33	42	125
272_2725805_2749883_24078	14	18	19	20	21
272_2798915_2881611_82696	28	35	70	102	116
272_302620_327391_24771	8	8	8	98	111
272_3101197_3186026_84829	32	43	51	70	75
272_3467282_3487820_20538	18	22	24	67	72
272_4549089_4563053_13964	2	46	54	74	80
272_4584587_4605128_20541	6	6	6	6	7
272_5076019_5097779_21760	27	34	37	47	52
272_894716_934619_39903	1	1	1	1	33
319_1216018_1265904_49886	10	10	10	88	100
319_1533717_1586902_53185	4	17	17	33	36
319_1600815_1647347_46532	9	9	9	9	10
319_1672122_1678249_6127	2	41	48	66	71
319_1737908_1853259_115351	29	36	40	55	60
319_2137516_2194922_57406	11	11	11	11	12
319_2532141_2557015_24874	15	19	20	21	22
319_2608045_2663185_55140	25	30	46	63	68
319_2781049_2805127_24078	14	18	19	34	37
319_2862921_2918181_55260	20	24	27	32	95

319_286987_312284_25297	8	16	16	16	17
319_3162469_3171021_8552	2	41	67	92	104
319_3448937_3468101_19164	18	22	24	27	108
319_4585655_4597486_11831	6	6	21	22	23
319_5076839_5098608_21769	27	34	37	51	56
319_885399_925301_39902	1	1	1	28	29
350_1227361_1273991_46630	10	10	45	62	67
350_1491588_1544772_53184	4	17	17	33	36
350_1558685_1605217_46532	9	9	9	9	10
350_1629992_1636119_6127	2	41	48	66	71
350_1695778_1762817_67039	24	29	71	104	118
350_2047082_2104487_57405	11	11	11	11	12
350_2441707_2466581_24874	15	19	20	21	22
350_2517611_2572414_54803	25	30	33	96	109
350_2600695_2660490_59795	3	3	3	3	4
350_2748143_2772209_24066	14	18	19	34	37
350_2829997_2885746_55749	20	24	27	32	35
350_302678_323631_20953	8	8	68	97	110
350_3131346_3139898_8552	2	41	67	92	104
350_3417926_3439735_21809	18	22	24	67	72
350_4520941_4541482_20541	6	6	6	6	7
350_5012437_5034204_21767	27	34	37	47	52
350_890946_930848_39902	1	1	1	28	29
472_1177033_1226234_49201	10	10	59	80	87
472_1489567_1548385_58818	4	4	4	4	5
472_1652771_1705691_52920	35	47	55	75	81
472_1874502_1980060_105558	13	51	60	81	89
472_2060500_2066626_6126	2	41	48	66	119
472_2090818_2135091_44273	9	9	9	54	59
472_2159089_2201462_42373	31	40	44	61	66
472_2537171_2563359_26188	15	19	20	21	22
472_2614328_2667799_53471	25	30	33	101	115
472_2698741_2760202_61461	3	13	65	87	96
472_2844779_2868845_24066	14	18	19	83	91
472_302608_327880_25272	8	8	38	94	106
472_3165947_3238821_72874	7	7	7	7	8
472_3520947_3541659_20712	18	22	24	67	139
472_4623805_4644347_20542	6	6	6	6	7
472_5115298_5137065_21767	27	34	37	60	65
472_896286_936188_39902	1	1	1	1	1
7784_1192290_1240648_48358	10	10	10	48	88
7784_1508626_1561824_53198	4	17	17	45	49
7784_1577050_1623011_45961	9	9	9	9	83
7784_1727088_1780238_53150	18	32	35	44	48
7784_2051486_2105426_53940	36	52	62	84	92
7784_2440962_2467150_26188	15	19	20	89	99

7784_2518139_2562038_43899	25	30	33	116	136
7784_2597657_2662969_65312	3	3	64	86	94
7784_2798875_2850123_51248	20	38	42	57	62
7784_293832_347971_54139	16	20	22	23	24
7784_3094432_3102984_8552	2	41	67	92	104
7784_3381129_3400431_19302	18	22	24	118	140
7784_4971004_4992771_21767	27	34	37	47	52
7784_5218103_5233576_15473	31	53	66	91	103
7784_921067_960965_39898	1	1	1	37	40
9000_1056331_1068060_11729	2	2	2	2	2
9000_1175283_1223041_47758	10	10	10	48	88
9000_1478585_1531781_53196	4	17	17	45	49
9000_1545694_1592227_46533	9	9	9	9	120
9000_1616239_1622366_6127	2	41	48	66	71
9000_1693246_1751239_57993	11	11	11	49	132
9000_2017305_2169911_152606	13	15	15	15	16
9000_2513170_2538037_24867	15	19	20	21	22
9000_2589006_2643800_54794	25	30	33	127	150
9000_2672081_2732608_60527	3	13	63	85	93
9000_2868857_2922113_53256	20	38	42	59	64
9000_302612_327398_24786	8	8	38	50	55
9000_3155803_3235122_79319	41	62	84	122	144
9000_3520504_3540857_20353	18	22	24	65	70
9000_4619511_4633252_13741	31	53	74	108	124
9000_5117130_5138897_21767	27	34	37	47	52
9000_896025_934610_38585	1	1	1	1	1
EC4115_1163585_1215779_52194	10	10	10	10	11
EC4115_1375525_1390585_15060	2	12	12	12	13
EC4115_1482236_1532758_50522	4	17	17	31	34
EC4115_1546671_1593200_46529	9	9	9	41	45
EC4115_1678881_1782574_103693	26	31	34	43	47
EC4115_2068013_2153419_85406	5	5	18	19	20
EC4115_2498400_2524587_26187	15	19	20	21	50
EC4115_2575618_2629114_53496	25	30	33	42	125
EC4115_2657393_2719919_62526	3	13	13	13	14
EC4115_2807572_2831650_24078	14	18	19	34	37
EC4115_2880663_2999662_118999	30	39	43	58	63
EC4115_302613_327404_24791	8	8	8	8	9
EC4115_3224208_3301441_77233	19	23	25	26	31
EC4115_3583765_3604425_20660	18	22	24	67	78
EC4115_5175376_5197136_21760	27	34	37	47	52
EC4115_894469_933059_38590	1	1	1	1	3
EDL933_1248355_1297334_48979	10	10	10	124	146
EDL933_1333375_1402824_69449	37	61	82	117	138
EDL933_1626386_1742105_115719	21	26	29	36	39
EDL933_1828825_1942373_113548	29	36	40	76	82

EDL933_2114833_2175910_61077	39	58	77	111	128
EDL933_2281971_2349347_67376	11	11	81	115	134
EDL933_2686568_2711439_24871	15	19	20	21	22
EDL933_2762470_2815951_53481	25	30	33	95	107
EDL933_291211_317714_26503	8	16	16	18	19
EDL933_2977093_3035748_58655	34	45	53	73	79
EDL933_3279965_3288517_8552	2	41	67	92	104
EDL933_3566546_3587810_21264	18	22	24	67	72
EDL933_4668424_4688965_20541	6	6	6	6	7
EDL933_890879_929465_38586	1	1	1	1	1
sakai_1161023_1209862_48839	10	10	10	48	53
sakai_1245903_1316397_70494	37	54	69	99	112
sakai_1431940_1446999_15059	2	12	12	12	13
sakai_1538645_1591830_53185	4	17	17	45	98
sakai_1586046_1621988_35942	33	44	52	71	76
sakai_1609996_1656529_46533	9	9	9	9	120
sakai_1757549_1814804_57255	18	37	41	56	61
sakai_1920485_1972367_51882	39	58	86	126	149
sakai_2153413_2254887_101474	23	33	36	46	51
sakai_2592106_2616977_24871	15	19	20	21	22
sakai_2668007_2712036_44029	25	30	46	100	113
sakai_2888774_2947429_58655	34	45	53	73	79
sakai_291210_317713_26503	8	16	16	18	19
sakai_3192960_3201512_8552	2	41	67	92	104
sakai_3479657_3500189_20532	18	22	24	67	72
sakai_4580800_4601341_20541	6	6	6	6	7
sakai_5042064_5079469_37405	40	60	79	113	131
sakai_891108_929694_38586	1	1	1	1	1
SS52_1164338_1214353_50015	10	10	10	107	123
SS52_1374507_1389567_15060	2	12	12	12	13
SS52_1481219_1531741_50522	4	17	17	31	34
SS52_1545654_1592183_46529	9	9	9	41	45
SS52_1691916_1752167_60251	22	27	30	38	41
SS52_2029150_2114560_85410	5	5	18	19	135
SS52_2459542_2485728_26186	15	19	20	21	50
SS52_2536756_2590249_53493	25	30	33	42	46
SS52_2618528_2681053_62525	3	13	13	13	148
SS52_2768706_2792784_24078	14	18	19	34	37
SS52_2841798_2914931_73133	30	50	58	123	145
SS52_302612_327288_24676	8	8	8	52	57
SS52_3139477_3216710_77233	19	23	25	26	27
SS52_3499038_3519658_20620	18	22	24	25	26
SS52_4600446_4620988_20542	6	6	6	6	7
SS52_5091916_5113676_21760	27	34	37	47	52
SS52_894355_932943_38588	1	1	1	1	3
TW14359_1165178_1216065_50887	10	10	10	10	11

TW14359_1375812_1390872_15060	2	12	12	12	13
TW14359_1482524_1533046_50522	4	17	17	31	34
TW14359_1546959_1593488_46529	9	9	9	41	45
TW14359_1691910_1789977_98067	22	42	49	68	73
TW14359_2066989_2152400_85411	5	5	18	19	20
TW14359_2497381_2523568_26187	15	19	20	21	50
TW14359_2574599_2628094_53495	25	30	33	96	130
TW14359_2656374_2718900_62526	3	13	13	13	14
TW14359_2806552_2830630_24078	14	18	19	20	21
TW14359_2880957_2954533_73576	30	50	58	79	86
TW14359_302613_328716_26103	8	8	8	8	43
TW14359_3179079_3256312_77233	19	23	25	26	31
TW14359_3538642_3559150_20508	18	22	24	67	72
TW14359_5124172_5153263_29091	27	34	37	72	77
TW14359_896062_934652_38590	1	1	1	1	3

Supplementary Figure 1: EasyFig alignments of t4.5 prophage clusters 1 (A), 9 (B), and 19 (C) from Fig. 3. The strain order for panel A is: 10671, 155, 180, 272, 319, 350, 472, 7784, 9000, EC4115, EDL933, Sakai, SS52, TW14359. For panel B: 10671, 155, 180, 272, 319, 350, 472, 7784, 9000, EC4115, Sakai, SS52, TW14359. For panel C: 10671, 155, 180, 272, 319, 350, 472, 7784, 9000, EC4115, EDL933, Sakai, SS52, TW14359.

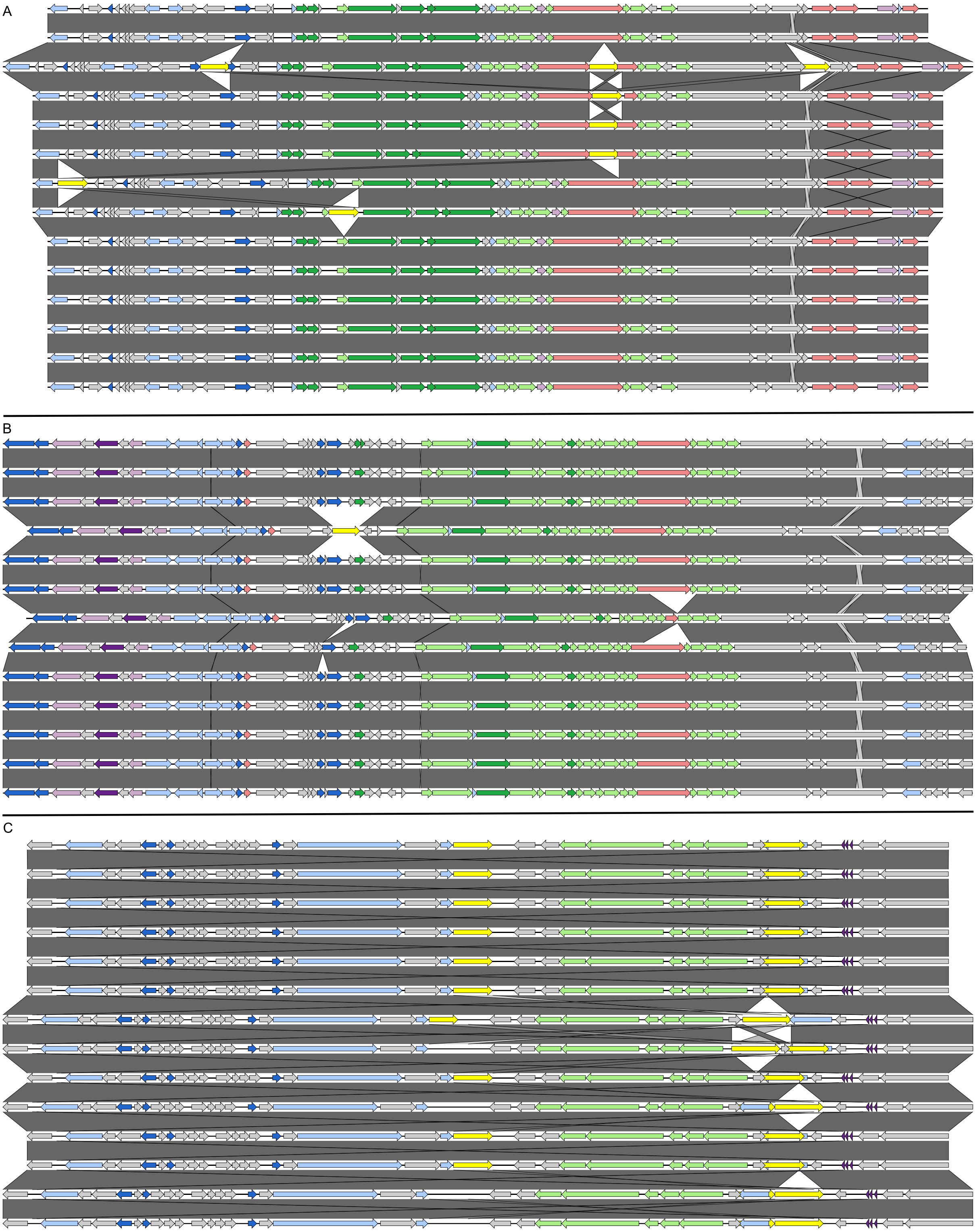

Supplementary Figure 2: Line plot showing the outputted numbers of homologues clusters by GetHomologues against the cut-offs used for these runs. The number of clusters rises as the cut-offs increase. However, this increase is only of ~60% when looking from the 5% to the 95% cut-off, and a ~40% when looking from the 40% to the 95% cut-off.

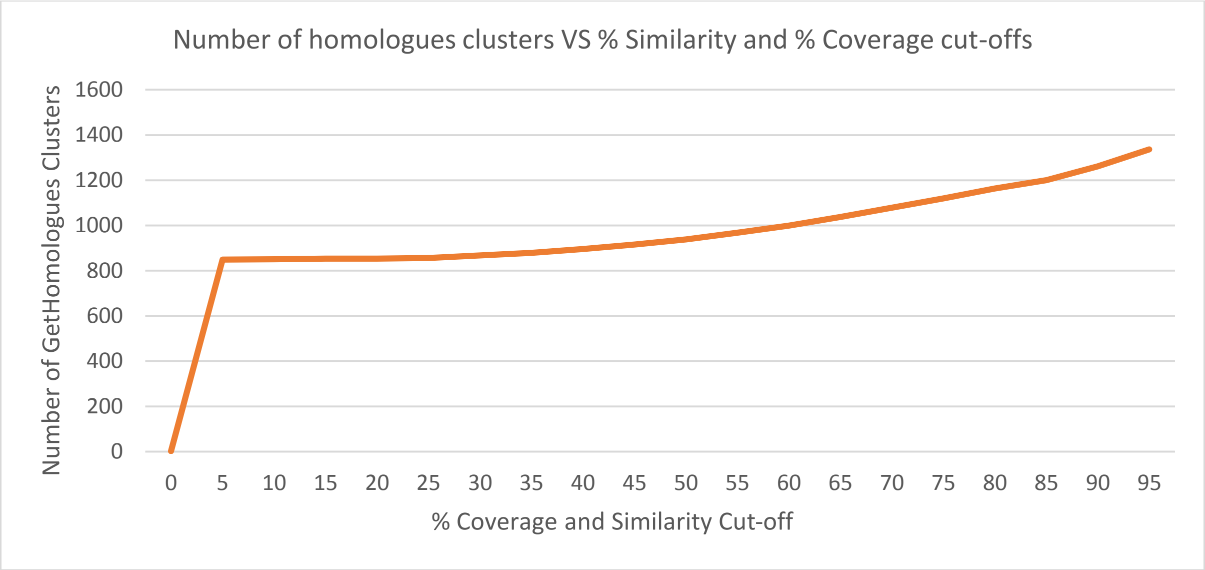

Supplement: Supplementary File 1 [file mgen-02-96-s001.pdf]
